# *Hyalosympodium* gen. et sp. nov. (*Pararamichloridiales*, *Diaporthomycetidae*) from freshwater habitats in Guizhou, China

**DOI:** 10.3897/mycokeys.136.195917

**Published:** 2026-07-06

**Authors:** Wangming Zhang, Zengjie Gao, Xiaoyu Song, Xinzhong Zhou, Wanqing Xie, Yuxi Yang, Juan Lu, Qinying Feng

**Affiliations:** 1 Beijing Jishuitan Hospital Guizhou Hospital, Guiyang 550014, China Beijing Jishuitan Hospital Guizhou Hospital Guiyang China; 2 Guizhou University of Traditional Chinese Medicine, Guiyang 550025, China Guizhou University of Traditional Chinese Medicine Guiyang China

**Keywords:** Asexual morph, lignicolous fungi, phylogeny, taxonomy

## Abstract

A novel genus, *Hyalosympodium*, is proposed to accommodate a lignicolous freshwater hyphomycete isolated from decaying wood collected in Guizhou Province, China. The taxon is established based on an integrative approach combining detailed morphological observations and multilocus phylogenetic analyses of LSU, ITS, SSU, and *rpb2* sequence data. Phylogenetically, *Hyalosympodium aquaticum* represents a monophyletic lineage within *Pararamichloridiales* and is resolved as sister to *Tenebrosynnematica obclavata*, with strong statistical support. Morphologically, *Hyalosympodium* is characterized by macronematous, synnematous, grouped, cylindrical, septate, subhyaline to brown conidiophores; polyblastic, integrated, sympodial, cylindrical, smooth, subhyaline to pale brown conidiogenous cells; and acropleurogenous, ellipsoidal to oval, guttulate, hyaline conidia. A comprehensive taxonomic treatment is provided, including detailed morphological descriptions, illustrations, and a phylogenetic tree to support the establishment of the new genus and species.

## Introduction

Aquatic fungi represent a highly diverse ecological group that plays a fundamental role in freshwater ecosystems, particularly in the decomposition of submerged plant debris and the regulation of nutrient cycling (Yuen et al. 1998; Bucher et al. 2004; Hyde et al. 2016a; Luo et al. 2019; Calabon et al. 2023). These fungi contribute significantly to organic matter turnover and energy flow in aquatic habitats, thereby sustaining ecosystem functioning. With the advent and widespread application of molecular phylogenetic approaches, traditional classifications based solely on morphology have been increasingly challenged and re-evaluated. Consequently, numerous taxa have undergone taxonomic revision, leading to the establishment of many novel genera and families, especially from freshwater environments in tropical and subtropical regions (Calabon et al. 2022; Yang et al. 2023; Liu et al. 2024; Ma et al. 2024; Wang et al. 2024a, 2024b, 2025; Shen et al. 2024; Lin et al. 2025; Xiao et al. 2025). In recent years, China has emerged as one of the most important centers of freshwater fungal diversity. In particular, regions such as Guangxi, Guizhou, Hainan, Hong Kong, and Yunnan have been recognized as biodiversity hotspots due to their unique climatic conditions and diverse freshwater habitats (Luo et al. 2004; Su et al. 2016; Dong et al. 2020; Bao et al. 2021, 2023; Yang et al. 2023; Shen et al. 2024). Extensive surveys conducted by various researchers in these areas have revealed a remarkable diversity of freshwater fungi, including a large number of previously undescribed taxa (Lu et al. 2018; Shen et al. 2022a, 2022b, 2024; Yang et al. 2023; Liu et al. 2024; Ma et al. 2024; Lin et al. 2025; Xiao et al. 2025). These findings not only expand the understanding of fungal diversity but also contribute to refining the taxonomic framework and phylogenetic relationships within freshwater fungal lineages.

Based on combined molecular phylogenetic evidence and morphological comparisons, the order *Pararamichloridiales* was established by Crous et al. (2017), with *Pararamichloridiaceae* designated as the type family. Subsequent phylogenetic and divergence-time analyses by Hyde et al. (2021) supported the circumscription of *Pararamichloridiales* as comprising the families *Pararamichloridiaceae*, *Neoeriomycopsidaceae*, and *Woswasiaceae*. Currently, the order *Pararamichloridiales* comprises seven genera: *Brunneoloconidia*, *Cyanoannulus*, *Neoeriomycopsis*, *Pararamichloridium*, *Tenebrosynnematica*, *Woswasia*, and *Xylochrysis* (Liu et al. 2024; Xiao and Liu 2025; Tan et al. 2026). Among these, *Brunneoloconidia* and *Tenebrosynnematica* are of uncertain family placement (*incertae sedis*) (Liu et al. 2024; Tan et al. 2026).

In this study, a novel lignicolous freshwater hyphomycete was collected from submerged decaying wood in freshwater habitats of Guizhou Province, China. Detailed morphological descriptions, combined with multilocus phylogenetic analyses based on LSU, ITS, SSU, and *rpb2* sequence data, revealed that this taxon represents a distinct and previously unrecognized lineage within *Diaporthomycetidae*. Based on morphological evidence and phylogenetic analyses, *Hyalosympodium* gen. nov. is introduced to accommodate this lineage, with *H. aquaticum* sp. nov. designated as the type species. This discovery further enriches the known diversity of lignicolous freshwater fungi in southwestern China and highlights the importance of integrating morphological and molecular data in resolving fungal taxonomy.

## Materials and methods

### Sample collection and specimen examination

Fungal samples were collected from a freshwater stream in Baiyun District, Guiyang City, Guizhou Province, China, on 12 October 2025. Samples were taken to the laboratory in plastic bags labeled with collection details, including locality, habitat, and date (Rathnayaka et al. 2025). Samples were cultured in plastic boxes lined with moistened tissue at room temperature for 1–2 weeks (Yang et al. 2023). The samples were examined using a stereomicroscope (SMZ 800, Nikon, Japan). Micromorphological characters were captured using an EOS 90D digital camera attached to an ECLIPSE Ni compound microscope (Nikon, Japan). Measurements of conidiophores, conidiogenous cells, and conidia were carried out using the Tarosoft® Image Framework program.

### Isolation and material deposition

Single-spore isolation was performed following the method described by Senanayake et al. (2020). The germinated conidia were aseptically transferred to fresh potato dextrose agar (PDA; Oxoid, CM0139) and incubated at room temperature for 39 days. Morphological characteristics of the fungal mycelium on PDA, including color, shape, size, margin, and elevation, were documented. Dried fungal specimens were deposited in the Herbarium of Guizhou Academy of Agricultural Sciences (Herb. GZAAS), Guiyang, China. Pure cultures were deposited at the Guizhou Culture Collection (GZCC), Guiyang, China. Descriptions of the new taxa were uploaded to the Faces of Fungi webpage following the guidelines of Jayasiri et al. (2015). The new species was registered in MycoBank.

### DNA extraction, PCR amplification, and sequencing

The mycelium, freshly scraped from living cultures, was transferred to 1.5 mL microcentrifuge tubes and stored in a refrigerator at −20 °C. Genomic DNA was extracted using the Biospin Fungus Genomic DNA Extraction Kit (BioFlux, China), following the manufacturer’s protocol. The primer pairs LR0R/LR5 (Vilgalys and Hester 1990), ITS5/ITS4 (White et al. 1990), NS1/NS4 (White et al. 1990), and fRPB2-5F/fRPB2-7cR (Liu et al. 1999) were used to amplify the LSU, ITS, SSU, and *rpb2* regions, respectively. PCR amplification was performed in a 25 μL reaction volume consisting of 13.5 μL of 2× Taq PCR Master Mix (China; containing Taq DNA polymerase, dNTPs, MgCl_2_, and reaction buffer), 1 μL of each primer, 1 μL of template DNA, and 8.5 μL of ddH_2_O. The polymerase chain reaction (PCR) conditions employed were in accordance with the reaction conditions outlined by Zhang et al. (2025). The PCR products were purified and sequenced by Sangon Biotech (Shanghai, China) Co., Ltd.

### Phylogenetic analyses

The forward and reverse primers of the newly generated sequences were checked and assembled using BioEdit v. 7.0.5.3 (Hall 1999) and SeqMan v. 7.0.0 (Swindell and Plasterer 1997). Similar taxa were identified through BLASTn searches conducted on the NCBI website (https://blast.ncbi.nlm.nih.gov/Blast.cgi). Multiple sequence alignments for each locus dataset were performed using MAFFT v. 7.473 (Katoh et al. 2019) and visually inspected in AliView (Larsson 2014). The LSU, ITS, SSU, and *rpb2* alignments were trimmed using trimAl v. 1.2rev59 (Capella-Gutiérrez et al. 2009) and subsequently merged using SequenceMatrix v. 1.7.8 (Vaidya et al. 2011).

Maximum likelihood (ML) analysis was conducted using the IQ-TREE web server (http://iqtree.cibiv.univie.ac.at/) based on the Bayesian information criterion (BIC) (Nguyen et al. 2015). The substitution model was automatically selected by the server. Bayesian inference (BI) analysis was performed using MrBayes on XSEDE (3.2.7a) via CIPRES Science Gateway (Swofford 2002; Ronquist et al. 2012). The aligned FASTA file was converted to NEXUS format using AliView (Daniel et al. 2010). The best-fit evolutionary model for each dataset was determined using MrModeltest v. 2.3.10 (Nylander et al. 2008). The GTR+I+G substitution model was selected for LSU, ITS, and *rpb2*, whereas the GTR+G model was chosen for SSU. The posterior probabilities (PP) were determined based on Bayesian Markov chain Monte Carlo (BMCMC) sampling (Huelsenbeck and Ronquist 2001). Four simultaneous Markov chains were run for 10^7^ generations, and trees were sampled every 1,000^th^ generation. The burn-in phase was set at 25%, and the remaining trees were used to calculate posterior probabilities. Phylogenetic trees were visualized using FigTree v. 1.4.4 and further edited in PowerPoint. The photoplate was made using Adobe Photoshop CS6 software (Adobe Systems, USA).

### Phylogenetic results

To determine the phylogenetic position of the two novel strains, a multilocus analysis was conducted using combined LSU, ITS, SSU, and *rpb2* sequences. The concatenated dataset included 3,549 characters (LSU: 1–849, ITS: 850–1,447, SSU: 1,448–2,481, and *rpb2*: 2,482–3,549) across 75 taxa. Phylogenetic relationships were inferred using both maximum likelihood (ML) and Bayesian inference (BI), which produced consistent tree topologies. The resulting multigene phylogeny indicates that the two isolates represent a previously undescribed genus within *Pararamichloridiales* (*Diaporthomycetidae*, *Sordariomycetes*). The isolates, GZCC 23-0558 and GZCC 23-0559, clustered as a sister lineage to *Tenebrosynnematica obclavata* (CZCC 24-0140), supported by 94% ML and 1.00 PP (Table 1).

**Table 1.** Taxa used in this study, along with their corresponding GenBank accession numbers.

Taxon	Strain	GenBank accessions				References
		LSU	ITS	SSU	*rpb*2	
*Acidothrix acidophila*	CBS 136259^T^	MH877622	NR_164513	N/A	N/A	Hujslova et al. (2014)
*Amplistroma caroliniana*	BEO 9923	FJ532377	N/A	N/A	N/A	Réblová and Winka (2001)
*Annulatascus velatisporus*	MFLUCC 16-1441	KY244031	KY320183	KY244032	N/A	Campbell and Shearer (2004)
*Annulusmagnus triseptatus*	A54 10A^T^	AY590286	N/A	N/A	N/A	Yen et al. (2021)
*Aquapteridospora lignicola*	MFLUCC 15-0377^T^	KU221018	N/A	N/A	N/A	Réblová et al. (2016)
*Aquimonospora tratensis*	MFLUCC 17-2133^T^	MK335797	NR_170743	MK335778	MK344654	Réblová et al. (2016)
*Ascitendus austriacus*	CBS 102665^T^	NG_056942	N/A	NG_061014	N/A	Zhang et al. (2017)
*Atractospora reticulata*	CBS 127884^T^	KT991660	N/A	N/A	KT991649	Réblová et al. (2015)
*Barbatosphaeria barbirostris*	CBS 121149	EF577059	N/A	KM492851	KM492903	Hyde et al. (2021)
*Barbatosphaeria neglecta*	CBS 127693^T^	KM492868	N/A	KM492856	N/A	Ferrer et al. (2012)
*Biflagellospora papillata*	KUMCC 19-0061	MW287756	N/A	N/A	N/A	Schoch et al. (2009)
*Brachysporium nigrum*	M.R. 1346	KT991662	N/A	KT991643	KT991652	Spatafora et al. (2006)
Brunneoloconidia aquatica	GZCC 23-0558^T^	PX585879	PX585877	N/A	PX512851	Tan et al. (2026)
Brunneoloconidia aquatica	GZCC 23-0559	PX585880	PX585878	N/A	PX512852	Tan et al. (2026)
*Bullimyces communis*	AF281-5^T^	JF775587	N/A	JF758619	N/A	Crous et al. (2017)
*Ceratolenta caudata*	CBS 125234^T^	JX066704	N/A	NG_061136	JX066699	Crous et al. (2015)
*Ceratosphaeria lampadophora*	SMH4822	AY346270	N/A	N/A	N/A	Crous et al. (2018)
*Commelinaceomyces aneilematis*	MAFF 246963	LC474617	LC474614	LC474620	LC474629	Hyde et al. (2016b)
*Coniochaeta acaciae*	MFLUCC 18-0776^T^	MT501618	N/A	MT498798	N/A	Crous et al. (2021)
*Coniochaeta aurantiaca*	CGMCC 3.22339^T^	OQ758134	NR_191236	N/A	N/A	Li et al. 2023
*Coniochaeta corticalis*	JKI-GP-23-050^T^	PV259250	PV272673	N/A	N/A	Crous et al. (2025)
*Conlarium duplumascospora*	CGMCC 3.14938^T^	JN936991	NR_138382	JN936987	N/A	Réblová et al. (2018)
*Cyanoannulus petersenii*	R044-1a^T^	AY316358	N/A	N/A	N/A	Raja et al. (2003)
*Cyanoannulus petersenii*	R044-1b	AY316359	N/A	N/A	N/A	Raja et al. (2003)
*Dictyosporella aquatica*	MD1302^T^	KT241022	N/A	KT241023	N/A	Liu et al. (2012)
*Distoseptispora fluminicola*	DLUCC 0391	MG979762	MG979755	N/A	N/A	Su et al. (2016)
*Fragosphaeria purpurea*	CBS 133.34	AB189154	N/A	AF096176	N/A	Klaubauf et al. (2014)
*Hyalosympodium aquaticum*	GZCC 27-27596^T^	PZ296715	PZ296707	N/A	N/A	In this study
*Hyalosympodium aquaticum*	GZCC 27-27597	PZ296716	PZ296708	N/A	N/A	In this study
*Jennwenomyces navicularis*	BCRC FU30872^T^	MT224909	MT224914	N/A	N/A	Klaubauf et al. (2014)
*Junewangia sphaerospora*	CGMCC 3.18655	KX033572	KU999981	KX033543	N/A	Klaubauf et al. (2014)
*Lanspora coronata*	AFTOL-ID 736	U46889	N/A	N/A	DQ470899	Klaubauf et al. (2014)
*Lylea dalbergiae*	CPC 38960^T^	MZ064481	MZ064424	N/A	N/A	Klaubauf et al. (2014)
*Magnaporthe poae*	M23	JF414883	N/A	N/A	N/A	Klaubauf et al. (2014)
*Marquandomyces marquandii*	CBS 182.27	EF468845	AY624193	EF468990	EF468942	Klaubauf et al. (2014)
*Myrmecridium iridis*	CPC 25084^T^	KR476777	KR476744	N/A	N/A	Hernández-Restrepo et al. (2015)
*Nakataea oryzae*	CBS 252.34^T^	MH867001	KM484862	N/A	N/A	Crous et al. (2013)
*Neoeriomycopsis aristata*	CBS 139913^T^	KR476776	KR476743	N/A	N/A	Crous et al. (2015)
*Neoeriomycopsis acutispora*	CBS 101302	MH874337	MH862730	N/A	N/A	Crous et al. (2023)
*Neoeriomycopsis fissistigmae*	CPC 45225^T^	OR717030	OR680775	N/A	N/A	Crous et al. (2023)
*Neoeriomycopsis sabal*	GZCC 21-0276^T^	PP621052	PP592428	PP627313	PP780232	Zhang et al. (2024)
*Neoeriomycopsis wadeae*	BRIP 71660a^T^	N/A	PP420203	N/A	N/A	Tan and Shivas (2024)
*Neomyrmecridium septatum*	CBS 145073^T^	NG_066289	NR_161133	N/A	MK047544	Klaubauf et al. (2014)
*Neospadicoides lignicola*	MFLUCC 17-2444^T^	MK849854	MK828702	MK828309	MN124532	Klaubauf et al. (2014)
Brunneoloconidia aquatica	GZCC 23-0558^T^	PX585879	PX585877	N/A	PX512851	Tan et al. (2026)
Brunneoloconidia aquatica	GZCC 23-0559	PX585880	PX585878	N/A	PX512852	Tan et al. (2026)
*Ophiostoma piliferum*	AFTOL-ID 910	DQ470955	N/A	DQ471003	DQ470905	Réblová and Štěpánek (2018)
*Paracapsulospora metroxyli*	MFLUCC 15-0250^T^	KX646364	KX669037	KX669035	N/A	Arzanlou et al. (2007)
*Paradiplococcium singulare*	FMR 10752^T^	KY853517	KY853456	N/A	N/A	Xia et al. (2017)
*Pararamichloridium aquisubtropicum*	GZCC 21-0668^T^	OM339434	OM339437	N/A	N/A	Crous et al. (2025)
*Pararamichloridium caricicola*	CBS 145069^T^	MK047488	NR_161130	N/A	N/A	Crous et al. (2025)
*Pararamichloridium livistonae*	CPC 32156^T^	MG386084	N/A	N/A	N/A	Hyde et al. (2018)
*Pararamichloridium ouropretoense*	COAD 3991^T^	PV664959	PV664933	N/A	N/A	Crous et al. (2025)
*Pararamichloridium verrucosum*	CBS 128.86^T^	MH873621	NR_156653	NG_065540	N/A	Crous et al. (2025)
*Phialemoniopsis ocularis*	UTHSC 05-2527	HE599266	HE599281	N/A	N/A	Xia et al. (2017)
*Phomatospora bellaminuta*	AFTOL-ID 766	FJ176857	N/A	FJ176803	FJ238345	Goh and Kuo (2020)
*Platytrachelon abietis*	CBS 125235^T^	JX066703	N/A	JX066707	JX066698	Ariyawansa et al. (2015)
*Pleurophragmium bambusinum*	MFLUCC 12-0850^T^	KU863149	KU940161	N/A	N/A	Luo et al. (2019)
*Polylobatispora deltoidea*	NBRC 106820	LC495605	LC495612	N/A	N/A	Réblová et al. (2016)
*Proliferophorum thailandicum*	MFLUCC 17-0293^T^	MK028343	MK028344	MK028345	N/A	Perdomo et al. (2013)
*Pseudoconlarium punctiforme*	GZCC 20-0009^T^	MN897833	MT002306	MN901116	N/A	Lutzoni et al. (2004)
*Pseudoproboscispora thailandensis*	MFLUCC 15-0989^T^	NG_059843	NR_152549	NG_063646	N/A	Spatafora et al. (2006)
*Rhamphoria delicatula*	CBS 132724	MH878338	N/A	N/A	N/A	Dong et al. (2021)
*Rhodoveronaea varioseptata*	CBS 431.88^T^	EU041870	EU041813	N/A	N/A	Liu et al. (2024)
*Rubellisphaeria abscondita*	CBS 132078^T^	KT991666	N/A	KT991646	KT991657	Réblová et al. (2014)
*Spadicoides bina*	CBS 137794	KY931824	KY931796	KY931881	KY931851	Zhang et al. (2011)
*Sporidesmiella hyalospermum*	MFLUCC 18-1312	MK849839	MK828688	N/A	MN124520	Huhndorf et al. (2004)
*Sporidesmium pyriformatum*	MFLUCC 15-0620^T^	KX710141	KX710146	N/A	MF135649	Hyde et al. (2016b)
*Tenebrosynnematica obclavata*	CZCC 24-0140^T^	PP657335	PP657293	N/A	PP887799	Tanaka et al. (2020)
*Thyridium vestitum*	AFTOL-ID 172	AY544671	N/A	AY544715	DQ470890	Sung et al. (2007)
*Woswasia atropurpurea*	CBS 133167	JX233658	JX233658	JX233658	JX233659	Jaklitsch et al. (2013)
*Xylochrysis aquatica*	CGMCC 3.23639^T^	ON949926	ON949924	ON949925	ON954070	Afshari et al. (2025)
*Xylochrysis chiangraiensis*	MFLUCC 24-0550^T^	PV072612	PQ800261	PV072702	N/A	Afshari et al. (2025)
*Xylochrysis guttulata*	CGMCC 3.27589^T^	PV264870	PV264867	PV264873	N/A	Xiao and Liu (2025)
*Xylochrysis hydei*	GMB-W 1199^T^	PV113468	PV113466	N/A	PV134362	Hongsanan et al. (2025)
*Xylochrysis lucida*	CBS 135996^T^	MH877601	KF747734	KF539912	KF539913	Hernández-Restrepo et al. (2017)
*Xylolentia brunneola*	PRA 13611^T^	MG600398	MG600394	N/A	MG600402	Dai et al. (2016)

Note: “^T^” indicates ex-type strains. Newly generated sequences are shaded in blue. “N/A” indicates the unavailable data in GenBank.

## Taxonomy

### Hyalosympodium

Taxon classificationFungiPararamichloridialesDiaporthomycetidae

W.M. Zhang & Q.Y. Feng gen. nov.

4D591CDB-F423-5263-9AAE-E20249131D6E

905415

#### Etymology.

“*Hyalosympodium*” refers to the presence of hyaline conidia and sympodial conidiogenous cells.

#### Description.

***Saprobic*** on decaying wood in a freshwater habitat. **Asexual morph**: Hyphomycetous. ***Colonies*** on natural substrate superficial, effuse, gregarious, with hyaline conidial masses at the apex. ***Mycelium*** partly superficial, consisting of branched, septate, smooth-walled, hyaline to pale brown hyphae. ***Conidiophores*** macronematous, synnematous, group, cylindrical, straight or slightly flexuous, septate, subhyaline to brown, percurrently proliferating from cut ends. ***Conidiogenous cells*** polyblastic, integrated, sympodial, cylindrical, smooth, subhyaline to pale brown. ***Conidia*** acropleurogenous, ellipsoidal to oval, apically rounded, with a narrowed frill-like, 0–2-septate, slightly constricted at the central septum, guttulate, hyaline, smooth-walled. **Sexual morph**: Undetermined.

#### Type species.

*Hyalosympodium aquaticum* W.M. Zhang & Q.Y. Feng.

#### Notes.

**Figure 1.**
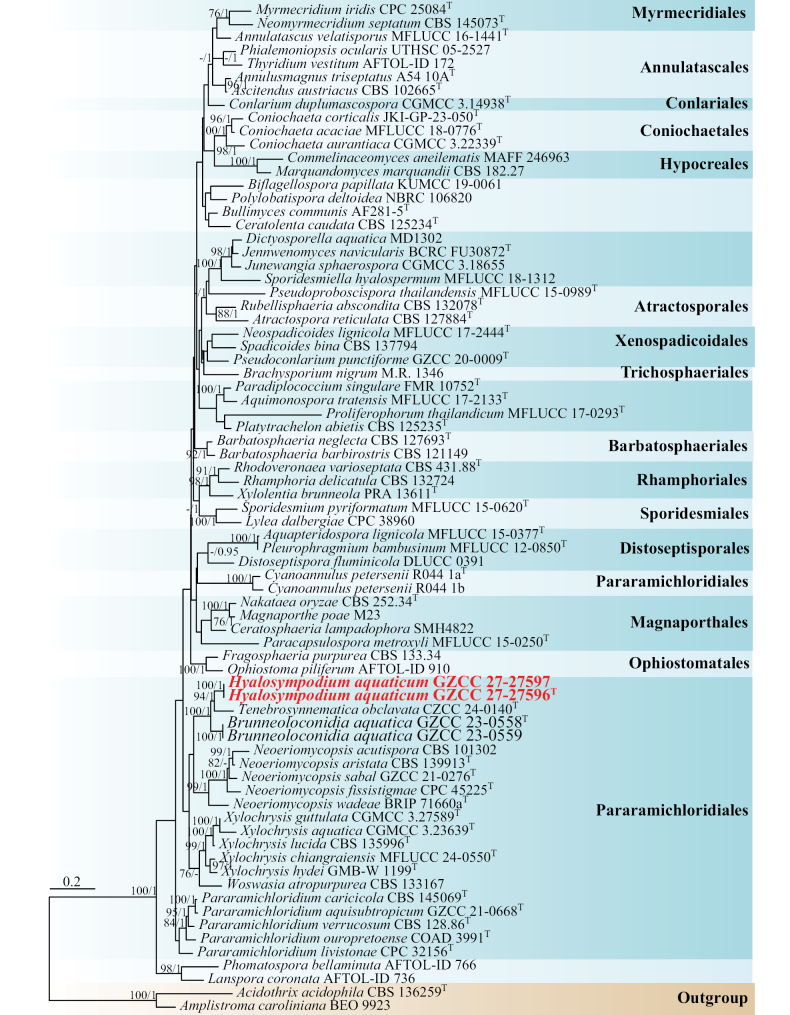
Phylogenetic tree inferred from maximum likelihood (ML) analysis based on a concatenated dataset of LSU, ITS, SSU, and *rpb2* sequences. Bootstrap support values ≥ 75% (ML, left) and Bayesian posterior probabilities ≥ 0.95 (PP, right) are indicated at the nodes. A hyphen (“-”) denotes support values < 75% for ML and < 0.95 for Bayesian inference (BI). The tree is rooted with *Acidothrix acidophila* (CBS 136259) and *Amplistroma caroliniana* (BEO 9923). Ex-type strains are indicated by “^T,^” and newly generated isolates are highlighted in bold red.

*Hyalosympodium* is proposed here to accommodate a lignicolous freshwater species collected from Guizhou Province, China. Phylogenetic analyses indicate that *H. aquaticum* forms a sister relationship with *Tenebrosynnematica obclavata* (CZCC 24-0140), with strong support (94% ML, 1.00 PP; Fig. 1). Morphologically, *Hyalosympodium* is distinguished from other *Pararamichloridiales* species by its distinct gregarious colonies and ellipsoidal to oval conidia (Raja et al. 2003; Jaklitsch et al. 2013; Crous et al. 2015; Hernández-Restrepo et al. 2017; Liu et al. 2024; Tan and Shivas 2024; Crous et al. 2025; Tan et al. 2026). Based on these combined morphological and phylogenetic data, the new genus *Hyalosympodium* (*Pararamichloridiales*, *Diaporthomycetidae*) is therefore established to accommodate *H. aquaticum*.

### Hyalosympodium aquaticum

Taxon classificationFungiPararamichloridialesDiaporthomycetidae

W.M. Zhang & Q.Y. Feng sp. nov.

5D13FDB4-A923-58F0-9CEF-E75EFC3B3238

905416

Fig. 2

#### Etymology.

“*aquaticum*’’ refers to the aquatic habitat of this fungus.

**Figure 2.**
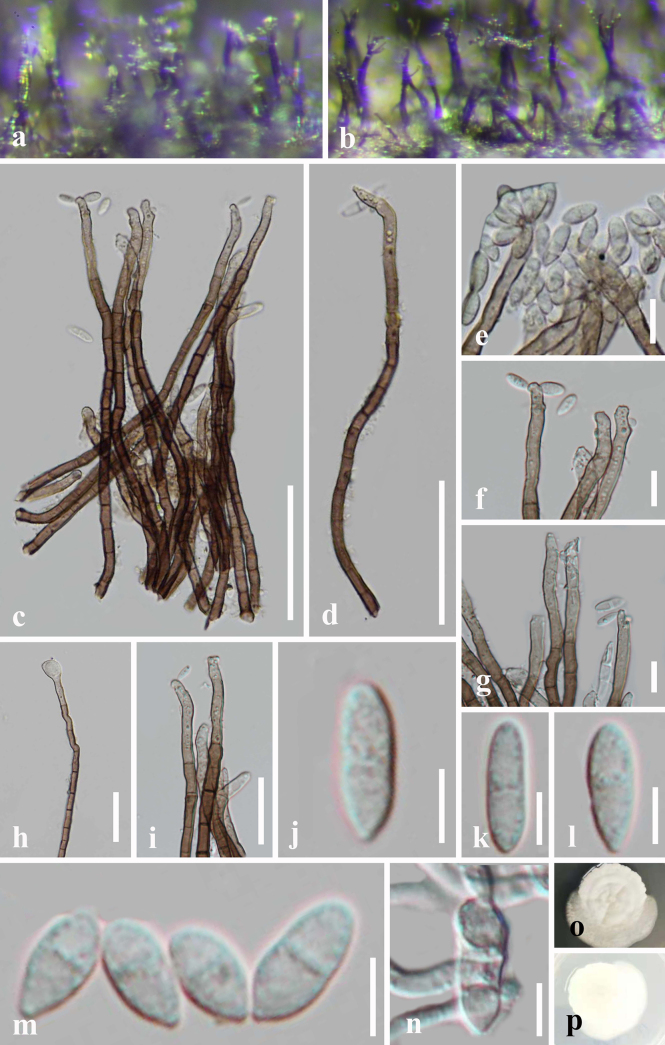
*Hyalosympodium aquaticum* (GZAAS 25-0787, holotype). **a, b**. Colonies on the host surface; **c, d**. Conidiophores, conidiogenous cells, and conidia; **e–i**. Conidiogenous cells and conidia; **j–m**. Conidia; **n**. Germinated conidium; **o, p**. Colonies on PDA from above and below after 39 days of incubation at room temperature. Scale bars: 50 μm (**c, d**); 30 μm (**h**); 20 μm (**i**); 10 μm (**e–g**); 5 μm (**j–n**).

#### Holotype.

GZAAS 25-0787.

#### Description.

***Saprobic*** on decaying wood in a freshwater habitat. **Asexual morph**: Hyphomycetous. ***Colonies*** on natural substrate superficial, effuse, gregarious, with hyaline conidial masses at the apex. ***Mycelium*** partly superficial, consisting of branched, septate, smooth-walled, hyaline to pale brown hyphae. ***Conidiophores*** 163–211 × 4–6 μm (x̄ = 183.5 × 5 μm, *n* = 30), macronematous, synnematous, group, cylindrical, straight or slightly flexuous, septate, subhyaline to brown, percurrently proliferating from cut ends. ***Conidiogenous cells*** 29–43 × 4–5 μm (x̄ = 34 × 4.5 μm, *n* = 30), polyblastic, integrated, sympodial, cylindrical, smooth, subhyaline to pale brown. ***Conidia*** 7–19 × 3–4.5 μm (x̄ = 10.5 × 4 μm, *n* = 35), acropleurogenous, ellipsoidal to oval, apically rounded, with a narrowed frill-like, 0–2-septate, slightly constricted at the central septum, guttulate, hyaline, smooth-walled. **Sexual morph**: Undetermined.

#### Culture characteristics.

Conidia germinate on PDA within 14 h, producing germ tubes from the conidial body. Colonies on PDA are irregular, with a flat surface and an undulate margin, reaching 33 mm in diameter after 39 days at 25 °C, and are white on both the surface and reverse.

#### Material examined.

China • Guizhou Province, Guiyang City, Baiyun District, Changpo Ling National Forest Park, on decaying wood in a freshwater habitat, 12 October 2025, Wang-Ming Zhang & Qinying Feng, XC6 (GZAAS 25-0787, holotype), ex-type living culture GZCC 27-27596; *ibid*., XC7(GZAAS 25-0788, paratype), living culture GZCC 27-27597.

#### Notes.

According to a BLASTn search on NCBI GenBank, the ITS and LSU sequences of the new isolate (GZCC 27-27596) share 85.79% similarity across 98% of the query sequence coverage and 96.27% similarity across 100% of the query sequence coverage with *T. obclavata* (CZCC 24-0140), respectively. In addition, base-pair comparisons between *H. aquaticum* (GZCC 27-27596) and *T. obclavata* (CZCC 24-0140) show 70/473 bp differences in ITS (14.8%, gaps 15 bp) and 12/813 bp differences in LSU (1.5%, gap of 1 bp). Morphologically, *H. aquaticum* (GZAAS 25-0787) can be readily distinguished from *T. obclavata* (GZAAS 24-0049) by its unique conidial morphology (ellipsoidal to oval, hyaline vs. fusiform to obclavate, brown) (Liu et al. 2024).

## Discussion

The combined evidence from molecular phylogenetic analyses and morphological characteristics supports the distinction of *Hyalosympodium* from *Brunneoloconidia* and *Tenebrosynnematica*. In the phylogenetic tree (Fig. 1), *B. aquatica*, *H. aquaticum*, and *T. obclavata* form a well-supported clade within the order *Pararamichloridiales*, which may represent a previously unrecognized family-level lineage. Morphologically, *Hyalosympodium* shares several features with *Brunneoloconidia*, including macronematous, cylindrical, septate conidiophores; polyblastic, integrated, sympodial, cylindrical conidiogenous cells; and acropleurogenous, guttulate, ellipsoidal, hyaline conidia (Tan et al. 2026). However, *Hyalosympodium* can be readily distinguished by its gregarious colonies, synnematous conidiophores, and hyaline, oval conidia, whereas *Brunneoloconidia* exhibits mononematous conidiophores, conidiogenous cells recurved at the apex, and fusiform, subhyaline to pale brown conidia (Tan et al. 2026). In addition, base-pair comparisons between *H. aquaticum* (ex-type GZCC 27-27596) and *B. aquatica* (ex-type GZCC 23-0558) reveal 77/541 bp differences in ITS (14.2%, including 14 gaps) and 32/858 bp differences in LSU (3.7%, including 2 gaps). Furthermore, species of *Brunneoloconidia*, *Hyalosympodium*, and *Tenebrosynnematica* differ morphologically from species in *Pararamichloridiaceae*, *Neoeriomycopsidaceae*, and *Woswasiaceae* by possessing pale brown to dark brown, elongated, septate, synnematous conidiophores and septate, hyaline to pale brown conidia (Raja et al. 2003; Jaklitsch et al. 2013; Crous et al. 2015; Hernández-Restrepo et al. 2017; Liu et al. 2024; Tan and Shivas 2024; Crous et al. 2025; Tan et al. 2026).

Liu et al. (2024) introduced the hyphomycetous genus *Tenebrosynnematica*, isolated from lignicolous terrestrial substrates in Guizhou, and assigned it to *Diaporthomycetidae* genera *incertae sedis*. Subsequently, Tan et al. (2026) described another hyphomycetous genus, *Brunneoloconidia*, from lignicolous freshwater substrates in Guizhou and, for the first time, placed both *Brunneoloconidia* and *Tenebrosynnematica* within the order *Pararamichloridiales*. In the present study, *Hyalosympodium* was similarly isolated and characterized from lignicolous freshwater substrates, further supporting the phylogenetic framework proposed by Tan et al. (2026). These findings underscore the value of integrating detailed morphological observations with multilocus phylogenetic analyses to clarify the relationships among hyphomycetous fungi in freshwater and terrestrial ecosystems.

In the present phylogenetic analyses, *Cyanoannulus petersenii* was not recovered within *Pararamichloridiales* but formed a separate lineage distant from members of the order. This finding raises questions regarding the monophyly of *Pararamichloridiales* and suggests that the order, as currently defined, may be polyphyletic. However, given the limited availability of sequence data for several related taxa and the insufficient taxon sampling across the group, this conclusion should be regarded as preliminary. The apparent polyphyly may also reflect the effects of morphological convergence among taxa currently assigned to the order. Therefore, additional molecular data and comprehensive phylogenetic studies are needed to reassess the circumscription and evolutionary relationships of *Pararamichloridiales*.

## Supplementary Material

XML Treatment for Hyalosympodium

XML Treatment for Hyalosympodium aquaticum

## Additional information

### Artificial Intelligence (AI) use

The authors accept full responsibility for the content of the manuscript, including the disclosure of any use of AI.

No AI tools were used in the preparation of this manuscript.

### Author ORCIDs

W. Zhang https://orcid.org/0009-0006-6967-8401

Z. Gao https://orcid.org/0000-0003-4289-1625

X. Song https://orcid.org/0000-0003-4152-6829

X. Zhou https://orcid.org/0009-0000-0459-1301

W. Xie https://orcid.org/0009-0001-0351-4248

Y. Yang https://orcid.org/0009-0006-5919-6513

J. Lu https://orcid.org/0009-0007-8962-1696

Q. Feng https://orcid.org/0000-0001-6635-7086

### Data availability

All of the data that support the findings of this study are available in the main text.

## References

[B1] Afshari N, Noorabadi MT, McKenzie EHC, Pumas C, Bhunjun CS, Jayawardena RS, Gomes de Farias AR, Phukhamsakda C, Jeewon R, Chaharmiri-Dokhaharani S, Suwannarach N, Kumla J, Al-Otibi F, Hyde KD, Lumyong S (2025) Taxonomy and diversity of woody litter microfungi associated with six phylogenetically related host species in Doi Tung national park Chiang Rai Thailand. Mycosphere 16(1): 4783–4935. 10.5943/mycosphere/16/1/36

[B2] Ariyawansa HA, Hyde KD, Jayasiri SC, Buyck B, Chen XH (2015) Fungal diversity notes 111–252 – taxonomic and phylogenetic contributions to fungal taxa. Fungal Diversity 75: 27–274. 10.1007/s13225-015-0346-5

[B3] Arzanlou M, Groenewald JZ, Gams W, Braun U, Crous PW (2007) Phylogenetic and morphotaxonomic revision of *Ramichloridium* and allied genera. Studies in Mycology 58: 57–93. 10.3114/sim.2007.58.03 PMC2104745 18490996

[B4] Bao DF, Hyde KD, McKenzie EHC, Jeewon R, Su HY, Nalumpang S, Luo ZL (2021) Biodiversity of lignicolous freshwater hyphomycetes from China and Thailand and description of sixteen species. Journal of Fungi 7(8): 669. 10.3390/jof7080669 PMC8399276 34436208

[B5] Bao DF, Hyde KD, Maharachchikumbura SSN, Perera RH, Thiyagaraja V, Hongsanan S, Wanasinghe DN, Shen HW, Tian XG, Yang LQ, Nalumpang S, Luo ZL (2023) Taxonomy, phylogeny and evolution of freshwater *Hypocreomycetidae (Sordariomycetes)*. Fungal Diversity 121: 1–94. 10.1007/s13225-023-00521-8

[B6] Bucher VVC, Hyde KD, Pointing SB, Reddy CA (2004) Production of wood decay enzymes, mass loss and lignin solubilization in wood by marine ascomycetes and their anamorphs. Fungal Diversity 15: 1–14.

[B7] Calabon MS, Hyde KD, Jones EBG, Luo ZL, Dong W, Hurdeal VG, Gentekaki E, Rossi W, Leonardi M, Thiyagaraja V, Lestari AS, Shen HW, Bao DF, Boonyuen N, Zeng M (2022) Freshwater fungal numbers. Fungal Diversity 114: 3–235. 10.1007/s13225-022-00503-2

[B8] Calabon MS, Hyde KD, Jones EBG, Bao DF, Bhunjun CS, Phukhamsakda C, Shen HW, Gentekaki E, Al Sharie AH, Barros J, Chandrasiri KSU, Hu DM, Hurdeal VG, Rossi W, Valle LG, Zhang H, Figueroa M, Raja HA, Seena S, Song HY, Dong W, El-Elimat T, Leonardi M, Li Y, Li YJ, Luo ZL, Ritter CD, Strongman DB, Wei MJ, Balasuriya A (2023) Freshwater fungal biology. Mycosphere 14: 195–413. 10.5943/mycosphere/14/1/4

[B9] Capella-Gutiérrez S, Silla-Martínez JM, Gabaldón T (2009) trimAl: a tool for automated alignment trimming in large-scale phylogenetic analyses. Bioinformatics 25(15): 1972–1973. 10.1093/bioinformatics/btp348 PMC2712344 19505945

[B10] Campbell J, Shearer CA (2004) *Annulusmagnus* and *Ascitendus*, two new genera in the *Annulatascaceae*. Mycologia 96(4): 822–833. 10.1080/15572536.2005.11832929 21148902

[B11] Crous PW, Wingfield MJ, Guarro J, Cheewangkoon R, van der Bank M, Swart WJ, Stchigel AM, Cano-Lira JF, Roux J, Madrid H, Damm U, Wood AR, Shuttleworth LA, Hodges CS, Munster M, de Jesus Yanez-Morales M, Zuniga-Estrada L, Cruywagen EM, de Hoog GS, Silvera C, Najafzadeh J, Davison EM, Davison PJ, Barrett MD, Barrett RL, Manamgoda DS, Minnis AM, Kleczewski NM, Flory SL, Castlebury LA, Clay K, Hyde KD, Mausse-Sitoe SN, Chen S, Lechat C, Hairaud M, Lesage-Meessen L, Pawlowska J, Wilk M, Sliwinska-Wyrzychowska A, Metrak M, Wrzosek M, Pavlic-Zupanc D, Maleme HM, Slippers B, Mac Cormack WP, Archuby DI, Grunwald NJ, Telleria MT, Duenas M, Martin MP, Marincowitz S, de Beer ZW, Perez CA, Gene J, Marin-Felix Y, Groenewald JZ (2013) Fungal planet description sheets: 154–213. Persoonia 31: 188–296. 10.3767/003158513X675925 PMC3904050 24761043

[B12] Crous PW, Wingfield MJ, Guarro J, Hernandez-Restrepo M, Sutton DA, Acharya K, Barber PA, Boekhout T, Dimitrov RA, Duenas M, Dutta AK, Gene J, Gouliamova DE, Groenewald M, Lombard L, Morozova OV, Sarkar J, Smith MT, Stchigel AM, Wiederhold NP, Alexandrova AV, Antelmi I, Armengol J, Barnes I, Cano-Lira JF, Castaneda Ruíz RF, Contu M, Courtecuisse PR, da Silveira AL, Decock CA, de Goes A, Edathodu J, Ercole E, Firmino AC, Fourie A, Fournier J, Furtado EL, Geering AD, Gershenzon J, Giraldo A, Gramaje D, Hammerbacher A, He XL, Haryadi D, Khemmuk W, Kovalenko AE, Krawczynski R, Laich F, Lechat C, Lopes UP, Madrid H, Malysheva EF, Marin-Felix Y, Martin MP, Mostert L, Nigro F, Pereira OL, Picillo B, Pinho DB, Popov ES, Rodas Pelaez CA, Rooney-Latham S, Sandoval-Denis M, Shivas RG, Silva V, Stoilova-Disheva MM, Telleria MT, Ullah C, Unsicker SB, van der Merwe NA, Vizzini A, Wagner HG, Wong PT, Wood AR, Groenewald JZ (2015) Fungal planet description sheets: 320–370. Persoonia 34: 167–266. 10.3767/003158513X675925 PMC4510277 26240451

[B13] Crous PW, Wingfield MJ, Burgess TI, Carnegie AJ, Hardy GESTJ, Smith D, Summerell BA, Cano-Lira JF, Guarro J, Houbraken J, Lombard L, Martín MP, Sandoval-Denis M, Alexandrova AV, Barnes CW, Baseia IG, Bezerra JDP, Guarnaccia V, May TW, Hernández-Restrepo M, Stchigel AM, Miller AN, Ordoñez ME, Abreu VP, Accioly T, Agnello C, Agustin Colmán A, Albuquerque CC, Alfredo DS, Alvarado P, Araújo-Magalhães GR, Arauzo S, Atkinson T, Barili A, Barreto RW, Bezerra JL, Cabral TS, Camello Rodríguez F, Cruz RHSF, Daniëls PP, da Silva BDB, de Almeida DAC, de Carvalho Júnior AA, Decock CA, Delgat L, Denman S, Dimitrov RA, Edwards J, Fedosova AG, Ferreira RJ, Firmino AL, Flores JA, García D, Gené J, Góis JS, Gomes AAM, Gonçalves CM, Gouliamova DE, Groenewald M, Guéorguiev BV, Guevara-Suarez M, Gusmão LFP, Hosaka K, Hubka V, Huhndorf SM, Jadan M, Jurjević Ž, Kraak B, Kučera V, Kumar TKA, Kušan I, Lacerda SR, Lamlertthon S, Lisboa WS, Loizides M, Luangsa-ard JJ, Lysková P, Mac Cormack WP, Macedo DM, Machado AR, Malysheva EF, Marinho P, Matočec N, Meijer M, Mešić A, Mongkolsamrit S, Moreira KA, Morozova OV, Nair KU, Nakamura N, Noisripoom W, Olariaga I, Oliveira RJV, Paiva LM, Pawar P, Pereira OL, Peterson SW, Prieto M, Rodríguez-Andrade E, Rojo De Blas C, Roy M, Santos ES, Sharma R, Silva GA, Souza-Motta CM, Takeuchi-Kaneko Y, Tanaka C, Thakur A, Smith MTH, Tkalčec Z, Valenzuela-Lopez N, Van der Kleij P, Verbeken A, Viana MG, Wang XW, Groenewald JZ (2017) Fungal planet description sheets: 625–715. Persoonia 39: 270–467. 10.3767/persoonia.2017.39.11 PMC5832955 29503478

[B14] Crous PW, Luangsa-ard JJ, Wingfield MJ, Carnegie AJ, Hernández-Restrepo M, Lombard L, Roux J, Barreto RW, Baseia IG, Cano-Lira JF, Martín MP, Morozova OV, Stchigel AM, Summerell BA, Brandrud TE, Dima B, García D, Giraldo A, Guarro J, Gusmão LFP, Khamsuntorn P, Noordeloos ME, Nuankaew S, Pinruan U, Rodríguez-Andrade E, Souza-Motta CM, Thangavel R, van Iperen AL, Abreu VP, Accioly T, Alves JL, Andrade JP, Bahram M, Baral H-O, Barbier E, Barnes CW, Bendiksen E, Bernard E, Bezerra JDP, Bezerra JL, Bizio E, Blair JE, Bulyonkova TM, Cabral TS, Caiafa MV, Cantillo T, Colmán AA, Conceição LB, Cruz S, Cunha AOB, Darveaux BA, da Silva AL, da Silva GA, da Silva GM, da Silva RMF, de Oliveira RJV, Oliveira RL, De Souza JT, Dueñas M, Evans HC, Epifani F, Felipe MTC, Fernández-López J, Ferreira BW, Figueiredo CN, Filippova NV, Flores JA, Gené J, Ghorbani G, Gibertoni TB, Glushakova AM, Healy R, Huhndorf SM, Iturrieta-González I, Javan-Nikkhah M, Juciano RF, Jurjević Ž, Kachalkin AV, Keochanpheng K, Krisai-Greilhuber I, Li YC, Lima AA, Machado AR, Madrid H, Magalhães OMC, Marbach PAS, Melanda GCS, Miller AN, Mongkolsamrit S, Nascimento RP, Oliveira TGL, Ordoñez ME, Orzes R, Palma MA, Pearce CJ, Pereira OL, Perrone G, Peterson SW, Pham THG, Piontelli E, Pordel A, Quijada L, Raja HA, Rosas de Paz E, Ryvarden L, Saitta A, Salcedo SS, Sandoval-Denis M, Santos TAB, Seifert KA, Silva BDB, Smith ME, Soares AM, Sommai S, Sousa JO, Suetrong S, Susca A, Tedersoo L, Telleria MT, Thanakitpipattana D, Valenzuela-Lopez N, Visagie CM, Zapata M, Groenewald JZ (2018) Fungal planet description sheets: 785–867. Persoonia 41: 238–417. 10.3767/persoonia.2018.41.12 PMC6344811 30728607

[B15] Crous PW, Cowan DA, Maggs-Kölling G, Yilmaz N, Thangavel R, Wingfield MJ, Noordeloos ME, Dima B, Brandrud TE, Jansen GM, Morozova OV, Vila J, Shivas RG, Tan YP, Bishop-Hurley S, Lacey E, Marney TS, Larsson E, Le Floch G, Lombard L, Nodet P, Hubka V, Alvarado P, Berraf-Tebbal A, Reyes JD, Delgado G, Eichmeier A, Jordal JB, Kachalkin AV, Kubátová A, Maciá-Vicente JG, Malysheva EF, Papp V, Rajeshkumar KC, Sharma A, Spetik M, Szabóová D, Tomashevskaya MA, Abad JA, Abad ZG, Alexandrova AV, Anand G, Arenas F, Ashtekar N, Balashov S, Bañares Á, Baroncelli R, Bera I, Biketova AYu, Blomquist CL, Boekhout T, Boertmann D, Bulyonkova TM, Burgess TI, Carnegie AJ, Cobo-Diaz JF, Corriol G, Cunnington JH, da Cruz MO, Damm U, Davoodian N, de A. Santiago ALCM, Dearnaley J, de Freitas LWS, Dhileepan K, Dimitrov R, Di Piazza S, Fatima S, Fuljer F, Galera H, Ghosh A, Giraldo A, Glushakova AM, Gorczak M, Gouliamova DE, Gramaje D, Groenewald M, Gunsch CK, Gutiérrez A, Holdom D, Houbraken J, Ismailov AB, Istel Ł, Iturriaga T, Jeppson M, Jurjević Ž, Kalinina LB, Kapitonov VI, Kautmanova I, Khalid AN, Kiran M, Kiss L, Kovács Á, Kurose D, Kusan I, Lad S, Læssøe T, Lee HB, Luangsa-ard JJ, Lynch M, Mahamedi AE, Malysheva VF, Mateos A, Matočec N, Mešić A, Miller AN, Mongkolsamrit S, Moreno G, Morte A, Mostowﬁzadeh-Ghalamfarsa R, Naseer A, Navarro-Ródenas A, Nguyen TTT, Noisripoom W, Ntandu JE, Nuytinck J, Ostrý V, Pankratov TA, Pawłowska J, Pecenka J, Pham THG, Polhorský A, Posta A, Raudabaugh DB, Reschke K, Rodríguez A, Romero M, Rooney-Latham S, Roux J, Sandoval-Denis M, Smith MTh, Steinrucken TV, Svetasheva TY, Tkalčec Z, van der Linde EJ, v.d. Vegte M, Vauras J, Verbeken A, Visagie CM, Vitelli JS, Volobuev SV, Weill A, Wrzosek M, Zmitrovich IV, Zvyagina EA, Groenewald JZ (2021) Fungal Planet description sheets: 1182–1283. Persoonia 46: 313–528. 10.3767/persoonia.2021.46.11 PMC9311394 35935893

[B16] Crous PW, Costa MM, Kandemir H, Vermaas M, Vu D, Zhao L, Arumugam E, Flakus A, Jurjević Ž, Kaliyaperumal M, Mahadevakumar S, Murugadoss R, Shivas RG, Tan YP, Wingfield MJ, Abell SE, Marney TS, Danteswari C, Darmostuk V, Denchev CM, Denchev TT, Etayo J, Gene J, Gunaseelan S, Hubka V, Illescas T, Jansen GM, Kezo K, Kumar S, Larsson E, Mufeeda KT, Piątek M, Rodriguez-Flakus P, Sarma PVSRN, Stryjak-Bogacka M, Torres-Garcia D, Vauras J, Acal DA, Akulov A, Alhudaib K, Asif M, Balashov S, Baral H-O, Baturo-Cieśniewska A, Begerow D, Beja-Pereira A, Bianchinotti MV, Bilański P, Chandranayaka S, Chellappan N, Cowan DA, Custódio FA, Czachura P, Delgado G, De Silva NI, Dijksterhuis J, Dueñas M, Eisvand P, Fachada V, Fournier J, Fritsche Y, Fuljer F, Ganga KGG, Guerra MP, Hansen K, Hywel-Jones N, Ismail AM, Jacobs CR, Jankowiak R, Karich A, Kemler M, Kisło K, Klofac W, Krisai-Greilhuber I, Latha KPD, Lebeuf R, Lopes ME, Lumyong S, Maciá-Vicente JG, Maggs-Kölling G, Magistà D, Manimohan P, Martín MP, Mazur E, Mehrabi-Koushki M, Miller AN, Mombert A, Ossowska EA, Patejuk K, Pereira OL, Piskorski S, Plaza M, Podile AR, Polhorský A, Pusz W, Raza M, Ruszkiewicz-Michalska M, Saba M, Sánchez RM, Singh R, Śliwa L, Smith ME, Stefenon VM, Strašiftáková D, Suwannarach N, Szczepańska K, Telleria MT, Tennakoon DS, Thines M, Thorn RG, Urbaniak J, van der Vegte M, Vasan V, Vila-Viçosa C, Voglmayr H, Wrzosek M, Zappelini J, Groenewald JZ (2023) Fungal Planet description sheets: 1550–1613. Persoonia 51: 280–417. 10.3767/persoonia.2023.51.08 PMC11041897 38665977

[B17] Crous PW, Catcheside DEA, Catcheside PS, Alfenas AC, Alfenas RF, Barreto RW, Lebel T, Balashov S, Broadbridge J, Jurjević Ž, De la Peña-Lastra S, Hoffmann R, Mateos A, Riebesehl J, Shivas RG, Soliz Santander FF, Tan YP, Altés A, Bandini D, Carriconde F, Cazabonne J, Czachura P, Gryta H, Eyssartier G, Larsson E, Pereira OL, Rigueiro-Rodríguez A, Wingfield MJ, Ahmad W, Bibi S, Denman S, Esteve-Raventós F, Hussain S, Illescas T, Luangsa-ard JJ, Möller L, Mombert A, Noisripoom W, Olariaga I, Pancorbo F, Paz A, Piątek M, Polman-Short C, Suárez E, Afshan NS, Ali H, Arzanlou M, Ayer F, Barratt J, Bellanger J-M, Bidaud A, Bishop-Hurley SL, Bohm M, Bose T, Campo E, Chau NB, Çolak ÖF, Cordeiro TRL, Cruz MO, Custódio FA, Couceiro A, Darmostuk V, Dearnaley JDW, de Azevedo Santiago ALCM, Freitas LWS, Yáñez-Morales MJ, Domnauer C, Dentinger B, Dhileepan K, De Souza JT, Dovana F, Eberhardt U, Eisvand P, Erhard A, Fachada V, García-Martín A, Groenewald M, Hammerbacher A, Harms K, Haroon S, Haqnawaz M, Henriques S, Hernández AJ, Jacobus LM, Jaen-Contreras D, Jangsantear P, Kaygusuz O, Knoppersen R, Kumar TKA, Lynch MJ, Mahiques R, Maraia GL, Marbach PAS, Mehrabi-Koushki M, Miller PR, Mongkolsamrit S, Moreau P-A, Oberlies NH, Oliveira JA, Orlovich D, Pérez-Méndez AS, Pinto A, Raja HA, Ramírez GH, Raphael B, Rodrigues A, Rodrigues H, Ramos DO, Safi A, Sarwar S, Saar I, Sánchez RM, Santana JS, Scrace J, Sales LS, Silva LNP, Stryjak-Bogacka M, Tacconi A, Thanh VN, Thomas A, Thuy NT, Toome M, Valdez-Carrazco JM, van Vuuren NI, Vasey J, Vauras J, Vila-Viçosa C, Villarreal M, Visagie CM, Vizzini A, Whiteside EJ, Groenewald JZ (2025) Fungal Planet description sheets: 1781–1866. Persoonia 54: 327–587. 10.3114/persoonia.2025.54.10 PMC12308287 40746709

[B18] Dai DQ, Phookamsak R, Wijayawardene NN, Li WJ, Bhat DJ, Xu JC, Taylor JE, Hyde KD, Chukeatirote E (2016) Bambusicolous fungi. Fungal Diversity 82: 1–105. 10.1007/s13225-016-0367-8

[B19] Daniel GP, Daniel GB, Miguel RJ, Florentino FR, David P (2010) ALTER: Program-oriented conversion of DNA and protein alignments. Nucleic Acids Research 38: W14–W18. 10.1093/nar/gkq321 PMC2896128 20439312

[B20] Dong W, Wang B, Hyde KD, McKenzie EHC, Raja HA, Tanaka K, Abdel-Wahab MA, Abdel-Aziz FA, Doilom M, Phookamsak R, Hongsanan S, Wanasinghe DN, Yu XD, Wang GN, Yang H, Yang J, Thambugala KM, Tian Q, Luo ZL, Yang JB, Miller AN, Fournier J, Boonmee S, Hu DM, Nalumpang S, Zhang H (2020) Freshwater *Dothideomycetes*. Fungal Diversity 105: 319–575. 10.1007/s13225-020-00463-5

[B21] Dong W, Hyde K, Jeewon R, Doilom M, Yu X, Wang G, Liu N, Hu D, Nalumpang S, Zhang H (2021) Towards a natural classification of *annulatascaceae*-like taxa II: introducing five new genera and eighteen new species from freshwater. Mycosphere 12: 1–88. 10.5943/mycosphere/12/1/1

[B22] Ferrer A, Miller AN, Sarmiento C, Shearer CA (2012) Three new genera representing novel lineages of *Sordariomycetidae* (*Sordariomycetes*, *Ascomycota*) from tropical freshwater habitats in Costa Rica. Mycologia 104(4): 865–879. 10.3852/11-111 22453118

[B23] Goh TK, Kuo CH (2020) *Jennwenomyces*, a new hyphomycete genus segregated from *Belemnospora*, producing versicolored phragmospores from percurrently extending conidiophores. Mycological Progress 19(9): 869–883. 10.1007/s11557-020-01602-7

[B24] Hall TA (1999) BioEdit: a user-friendly biological sequence alignment editor and analysis program for Windows 95/98/NT. Nucleic Acids Symposium Series 41: 95–98.

[B25] Hernández-Restrepo M, Groenewald JZ, Crous PW (2015) *Neocordana* gen. nov., the causal organism of *Cordana* leaf spot of banana. Phytotaxa 205(4): 229–238. 10.11646/phytotaxa.205.4.2

[B26] Hernández-Restrepo M, Gené J, Castañeda Ruíz RF, Mena-Portales J, Crous PW, Guarro J (2017) Phylogeny of saprobic microfungi from Southern Europe. Studies in Mycology 86: 53–97. 10.1016/j.simyco.2017.05.002 PMC5470572 28626275

[B27] Hongsanan S, Khuna S, Manawasinghe IS, Tibpromma S, Chethana KWT, Xie N, Bagacay JFE, Calabon MS, Chen C, Doilom M, Du HY, Gafforov Y, Huang SK, Li JX, Luangharn T, Luo ZL, Opiña LAD, Pem D, Sadaba RB, Singh R, Tan Q, Tang SM, Wang WP, Wen TC, Xia G, Zhao Q, Bhunjun CS, Cao B, Chen YP, de Silva NI, Dai DQ, Dong W8, Du TY, Ferreira-Sá AS, Gao Y, Gui H, Han LS, Han MY, Han XX, Jayawardena RS, Khyaju S, Kumar S, Lei L, Leonardo-Silva L, Li H, Li YX, Liao CF, Liu JW, Liu XF, Lu L, Lu WH, Luo M, Maharachchikumbura SSN, Meng QF, Mi LX, Norphanphoun C, Peng XC, Su HL, Tennakoon DS, Thiyagaraja V, Tun ZL, Wijayawardene NN, Xavier-Santos S, Xiong YR, Xu RF, Yadav S, Yang T, Yang YH, Yarasheva M, Zeng XY, Zhang H, Zhang GQ, Zhang X, Zhao HJ, Zhao RL, Zheng DG, Wanasinghe DN, Karunarathna SC (2025) Mycosphere Notes 521–571: A special edition of fungal biodiversity to celebrate Kevin D. Hyde’s 70^th^ birthday and his exceptional contributions to Mycology. Mycosphere 16(2): 1–178. 10.5943/mycosphere/16/2/1

[B28] Huelsenbeck JP, Ronquist FJB (2001) MRBAYES: Bayesian inference of phylogenetic trees. Bioinformatics 17: 754–755. 10.1093/bioinformatics/17.8.754 11524383

[B29] Huhndorf SM, Miller AN, Fernandez FA (2004) Molecular systematics of the *Sordariales*: the order and the family *Lasiosphaeriaceae* redefined. Mycologia 96(2): 368–387. 10.1080/15572536.2005.11832982 21148859

[B30] Hujslova M, Kubatova A, Kostovcik M, Blanchette RA, deBeer W, Chudicková M, Kolarik M (2014) The three new fungal genera from extremely acidic soils in the Czech Republic. Mycological Progress 13(3): 819–831. 10.1007/s11557-014-0965-3

[B31] Hyde KD, Fryar S, Tian Q, Bahkali AH, Xu J (2016a) Lignicolous freshwater fungi along a north–south latitudinal gradient in the Asian/Australian region; can we predict the impact of global warming on biodiversity and function? Fungal Ecology 19: 190–200. 10.1016/j.funeco.2015.07.002

[B32] Hyde KD, Hongsanan S, Jeewon R, Bhat DJ, McKenzie EHC, Jones EBG, Phookamsak R, Ariyawansa HA, Boonmee S, Zhao Q, Abdel-Aziz FA, Abdel-Wahab MA, Banmai S, Chomnunti P, Cui BK, Daranagama DA, Das K, Dayarathne MC, de Silva NI, Dissanayake AJ, Doilom M, Ekanayaka AH, Gibertoni TB, Go ´es-Neto A, Huang SK, Jayasiri SC, Jayawardena RS, Konta S, Lee HB, Li WJ, Lin CG, Liu JK, Lu YZ, Luo ZL, Manawasinghe IS, Manimohan P, Mapook A, Niskanen T, Norphanphoun C, Papizadeh M, Perera RH, Phukhamsakda C, Richter C, Santiago ALCMA, Drechsler-Santos ER, Senanayake IC, Tanaka K (2016b) Fungal diversity notes 367–491: taxonomic and phylogenetic contributions to fungal taxa. Fungal Diversity 80: 1–270. 10.1007/s13225-017-0378-0

[B33] Hyde KD, Chaiwan N, Norphanphoun C, Boonmee S, Camporesi E, Chethana KWT, Dayarathne MC, de Silva NI, Dissanayake AJ, Ekanayaka AH, Hongsanan S, Huang SK, Jayasiri SC, Jayawardena RS, Jiang HB, Karunarathna A, Lin CG, Liu JK, Liu NG, Lu YZ, Luo ZL, Maharachchimbura SSN, Manawasinghe IS, Pem D, Perera RH, Phukhamsakda C, Samarakoon MC, Senwanna C, Shang QJ, Tennakoon DS, Thambugala KM, Tibpromma S, Wanasinghe DN, Xiao YP, Yang J, Zeng XY, Zhang JF, Zhang SN, Bulgakov TS, Bhat DJ, Cheewangkoon R, Goh TK, Jones EBG, Kang JC, Jeewon R, Liu ZY, Lumyong S, Kuo CH, McKenzie EHC, Wen TC, Yan JY, Zhao Q (2018) Mycosphere notes 169–224. Mycosphere 9(2): 271–430. 10.5943/mycosphere/9/2/8

[B34] Hyde KD, Bao DF, Hongsanan S, Chethana KWT, Yang J, Suwannarach N (2021) Evolution of freshwater *Diaporthomycetidae (Sordariomycetes)* provides evidence for five new orders and six new families. Fungal Diversity 107: 71–105. 10.1007/s13225-021-00469-7

[B35] Jaklitsch WM, Réblová M, Voglmayr H (2013) Molecular systematics of *Woswasia atropurpurea* gen. et sp. nov. (*Sordariomycetidae*), a fungicolous ascomycete with globose ascospores and holoblastic conidiogenesis. Mycologia 105(2): 476–485. 10.3852/12-244 23099520

[B36] Jayasiri SC, Hyde KD, Ariyawansa HA, Bhat DJ, Buyck B, Cai L, Dai YC, Abd-Elsalam KA, Ertz D, Hidayat I (2015) The faces of fungi database: fungal names linked with morphology, phylogeny and human impacts. Fungal Diversity 74: 3–18. 10.1007/s13225-015-0351-8

[B37] Katoh K, Rozewicki J, Yamada KD (2019) MAFFT online service: multiple sequence alignment, interactive sequence choice and visualization. Briefings in Bioinformatics 20: 1160–1166. 10.1093/bib/bbx108 PMC6781576 28968734

[B38] Klaubauf S, Tharreau D, Fournier E, Groenewald JZ, Crous PW, De Vries RP, Lebrun MH (2014) Resolving the polyphyletic nature of *Pyricularia (Pyriculariaceae)*. Studies in Mycology 79: 85–120. 10.1016/j.simyco.2014.09.004 PMC4255532 25492987

[B39] Larsson A (2014) AliView: a fast and lightweight alignment viewer and editor for large datasets. Bioinformatics 30(22): 3276–3278. 10.1093/bioinformatics/btu531 PMC4221126 25095880

[B40] Li M, Raza M, Song S, Hou LW, Zhang ZF, Gao M, Huang JE, Liu F, Cai L (2023) Application of culturomics in fungal isolation from mangrove sediments. Add. file 1: Descriptions of novel taxa proposed in this study. Microbiome 11(272): 1–87 [Add. file 1]. 10.1186/s40168-023-01708-6 PMC10712113 38082427

[B41] Lin CG, Hyde KD, Feng Y, Xiao YP, Liu NG, Lu YZ, Luo ZL, Liu JK (2025) Notes, outline, systematics and phylogeny of hyaline-spored hyphomycetes. Fungal Diversity 135: 57–467. 10.1007/s13225-025-00561-2

[B42] Liu F, Hu DM, Cai L (2012) *Conlarium duplumascospora* gen. et. sp. nov. and *Jobellisia guangdongensis* sp. nov. from freshwater habitats in China. Mycologia 104(5): 1178–1186. 10.3852/11-379 22505431

[B43] Liu NG, Hyde KD, Sun YR, Bhat DJ, Jones EBG, Jumpathong JJ, Lin CG, Lu YZ, Yang J, Liu JJ, Liu ZY, Liu JK (2024) Notes, outline, taxonomy and phylogeny of brown-spored hyphomycetes. Fungal Diversity 129: 1–281. 10.1007/s13225-024-00539-6

[B44] Liu YJ, Whelen S, Hall BD (1999) Phylogenetic relationships among ascomycetes: evidence from an RNA polymerse II subunit. Molecular Biology and Evolution 16(12): 1799–1808. 10.1093/oxfordjournals.molbev.a026092 10605121

[B45] Lu YZ, Liu JK, Hyde KD, Jeewon R, Kang JC, Fan C, Boonmee S, Bhat DJ, Luo ZL, Lin CG (2018) A taxonomic reassessment of *Tubeufiales* based on multi-locus phylogeny and morphology. Fungal Diversity 92: 131–344. 10.1007/s13225-018-0411-y

[B46] Luo J, Yin J, Cai L, Zhang K, Hyde K (2004) Freshwater fungi in Lake Dianchi, a heavily polluted lake in Yunnan, China. Fungal Diversity 16: 93–112.

[B47] Luo ZL, Hyde KD, Liu JK, Maharachchikumbura SSN, Jeewon R, Bao DF, Bhat DJ, Lin CG, Li WL, Yang J, Liu NG, Lu YZ, Jayawardena RS, Li JF, Su HY (2019) Freshwater *Sordariomycetes*. Fungal Diversity 99: 451–660. 10.1007/s13225-019-00438-1

[B48] Lutzoni F, Kauff F, Cox CJ, McLaughlin D, Celio G, Dentinger B, Padamsee M, Hibbett D, James TY, Baloch E, Grube M, Reeb V, Hofstetter V, Schoch C, Arnold AE, Miadlikowska J, Spatafora J, Johnson D, Hambleton S, Crockett M, Shoemaker R, Sung GH, Lucking R, Lumbsch T, O’Donnell K, Binder M, Diederich P, Ertz D, Gueidan C, Hansen K, Harris RC, Hosaka K, Lim YW, Matheny B, Nishida H, Pfister D, Rogers J, Rossman A, Schmitt I, Sipman H, Stone J, Sugiyama J, Yahr R, Vilgalys R (2004) Assembling the fungal tree of life: progress classification and evolution of subcellular traits. American Journal of Botany 91(10): 1446–1480. 10.3732/ajb.91.10.1446 21652303

[B49] Ma J, Hyde KD, Tibpromma S, Gomdola D, Liu NG, Norphanphoun C, Bao DF, Boonmee S, Xiao XJ, Zhang LJ, Luo ZL, Zhao Q, Suwannarach N, Karunarathna SC, Liu JK, Lu YZ (2024) Taxonomy and systematics of lignicolous helicosporous hyphomycetes. Fungal Diversity 129: 365–653. 10.1007/s13225-024-00544-9

[B50] Nguyen LT, Schmidt HA, Von Haeseler A, Minh BQ (2015) IQ-TREE: a fast and effective stochastic algorithm for estimating maximum-likelihood phylogenies. Molecular Biology and Evolution 32(1): 268–274. 10.1093/molbev/msu300 PMC4271533 25371430

[B51] Nylander JAA, Zoology S, Posada D, Mrmodeltest R, Os F (2008) MrModeltest2 v. 2.3 (Program for Selecting DNA Substitution Models Using PAUP*). Evolutionary Biology Centre, Uppsala, Sweden.

[B52] Perdomo H, García D, Gené J, Cano J, Sutton DA, Summerbell R, Guarro J (2013) *Phialemoniopsis*, a new genus of *Sordariomycetes*, and new species of *Phialemonium* and *Lecythophora*. Mycologia 105(2): 398–421. 10.3852/12-137 23099515

[B53] Raja HA, Campbell J, Shearer CA (2003) Freshwater ascomycetes: *Cyanoannulus petersenii*, a new genus and species from submerged wood. Mycotaxon 88: 1–17. 10.5962/p.418455

[B54] Rathnayaka AR, Tennakoon DS, Jones GE, Wanasinghe DN, Bhat DJ, Priyashantha AH, Stephenson SL, Tibpromma S, Karunarathna SC (2025) Significance of precise documentation of hosts and geospatial data of fungal collections, with an emphasis on plant-associated fungi. New Zealand Journal of Botany 63(2–3): 462–489. 10.1080/0028825X.2024.2381734

[B55] Réblová M, Štěpánek V (2018) Introducing the *Rhamphoriaceae*, fam. nov. (*Sordariomycetes*), two new genera, and new life histories for taxa with *Phaeoisaria*- and *Idriella*-like anamorphs. Mycologia 11(4): 750–770. 10.1080/00275514.2018.1475164 30125239

[B56] Réblová M, Winka K (2001) Generic concepts and correlations in ascomycetes based on molecular and morphological data: *Lecythothecium duriligni* gen. et sp. nov. with a *Sporidesmium anamorph*, and *Ascolacicola austriaca* sp. nov. Mycologia 93(3): 478–493. 10.1080/00275514.2001.12063181

[B57] Réblová M, Štěpánek V, Schumacher RK (2014) *Xylochrysis lucida* gen. et sp. nov., a new lignicolous ascomycete (*Sordariomycetidae*) with holoblastic conidiogenesis. Mycologia 106(3): 564–572. 10.3852/13-266 24871596

[B58] Réblová M, Réblová K, Štěpánek V (2015) Molecular systematics of *Barbatosphaeria (Sordariomycetes)*: multigene phylogeny and secondary ITS structure. Persoonia 35: 21–38. 10.3767/003158515X687434 PMC4713105 26823626

[B59] Réblová M, Fournier J, Stepánek V (2016) Two new lineages of aquatic ascomycetes: *Atractospora* gen. nov. and *Rubellisphaeria* gen. et sp. nov., and a sexual morph of *Myrmecridium montsegurinum* sp. nov. Mycological Progress 15(21): 1–18. 10.1007/s11557-016-1166-z

[B60] Réblová M, Miller AN, Réblová K, Štěpánek V (2018) Phylogenetic classification and generic delineation of *Calyptosphaeria* gen. nov., *Lentomitella*, *Spadicoides* and *Torrentispora (Sordariomycetes)*. Studies in Mycology 89: 1–62. 10.1016/j.simyco.2017.11.004 PMC5773705 29367793

[B61] Ronquist F, Teslenko M, Van Der Mark P, Ayres DL, Darling A, Höhna S, Larget B, Liu L, Suchard MA, Huelsenbeck JP (2012) MrBayes 3.2: Efficient Bayesian Phylogenetic Inference and model choice across a large model space. Systematic Biology 61(3): 539–542. 10.1093/sysbio/sys029 PMC3329765 22357727

[B62] Schoch CL, Sung GH, Francesc López-Giráldez TJP, Spatafora JW (2009) The *Ascomycota* tree of life: a phylum-wide phylogeny clarifies the origin and evolution of fundamental reproductive and ecological traits. Systematic Biology 58(2): 224–239. 10.1093/sysbio/syp020 20525580

[B63] Senanayake IC, Rathnayaka AR, Marasinghe DS, Calabon MS, Gentekaki E, Lee HB, Hurdeal VG, Pem D, Dissanayake LS, Wijesinghe SN, Bundhun D, Nguyen TT, Goonasekara ID, Abeywickrama PD, Bhunjun CS, Jayawardena RS, Wanasinghe DN, Jeewon R, Bhat DJ, Xiang MM (2020) Morphological approaches in studying fungi: collection, examination, isolation, sporulation and preservation. Mycosphere 11(1): 2678–2754. 10.5943/mycosphere/11/1/20

[B64] Shen HW, Bao DF, Wanasinghe DN, Boonmee S, Liu JK, Luo ZL (2022a) Novel species and records of *Dictyosporiaceae* from freshwater habitats in China and Thailand. Journal of Fungi 8(11): 1200. 10.3390/jof8111200 PMC9694895 36422021

[B65] Shen HW, Bao DF, Bhat DJ, Su HY, Luo ZL (2022b) Lignicolous freshwater fungi in Yunnan Province, China: an overview. Mycology 13: 119–132. 10.1080/21501203.2022.2058638 PMC9196657 35711328

[B66] Shen HW, Luo ZL, Bao DF, Luan S, Bhat DJ, Boonmee S, Wang WP, Su XJ, Li YX, Al-Otibi F, Lu YZ, Yang LQ, Hyde KD (2024) Lignicolous freshwater fungi from China IV: Morphology and phylogeny reveal new species of *Pleosporales* from plateau lakes in Yunnan Province, China. Mycosphere 15(1): 6439–6524. 10.5943/mycosphere/15/1/28

[B67] Spatafora JW, Sung GH, Johnson D, Hesse C, O’Rourke B, Serdani M, Spotts R, Lutzoni F, Hofstetter V, Miadlikowska J, Reeb V, Gueidan C, Fraker E, Lumbsch T, Lucking R, Schmitt I, Hosaka K, Aptroot A, Roux C, Miller AN, Geiser DM, Hafellner J, Hestmark G, Arnold E, Budel B, Rauhut A, Hewitt D, Untereiner WA, Cole MS, ScheideggerC, Schultz M, Sipman H, Schoch CL (2006) A five-gene phylogeny of *Pezizomycotina*. Mycologia 98(6): 1018–1028. 10.3852/mycologia.98.6.1018 17486977

[B68] Su HY, Hyde KD, Maharachchikumbura SSN, Ariyawansa HA, Luo ZL, Promputtha I, Tian Q, Lin CG, Shang QJ, Zhao YC, Chai HM, Liu XY, Bahkali AH, Bhat JD, McKenzie EHC, Zhou DQ (2016) The families *Distoseptisporaceae* fam. nov., *Kirschsteiniotheliaceae*, *Sporormiaceae* and *Torulaceae*, with new species from freshwater in Yunnan Province, China. Fungal Diversity 80: 375–409. 10.1007/s13225-016-0362-0

[B69] Sung GH, Hywel-Jones NL, Sung JM, Luangsa-ard JJ, Shrestha B, Spatafora JW (2007) Phylogenetic classification of *Cordyceps* and the clavicipitaceous fungi. Studies in Mycology 57: 5–59. 10.3114/sim.2007.57.01 PMC2104736 18490993

[B70] Swindell SR, Plasterer TN (1997) Seqman. In Sequence Data Analysis Guidebook. Springer, Berlin, 75–89. 10.1385/0-89603-358-9:75

[B71] Swofford DL (2002) PAUP*: Phylogenetic analysis using parsimony (and other methods), Version 4.0 b10. MA, Sinauer Associates, Sunderland, UK.

[B72] Tan TH, Gao F, Wu CF, Zhao NN, Li HB, Kang GP, Ma J (2026) Morphological and multi-locus phylogenetic analyses reveal *Brunneoloconidia* gen. et sp. nov. (*Pararamichloridiales*, *Diaporthomycetidae*) from freshwater habitats in Guizhou Province, China. MycoKeys Submitted. 10.3897/mycokeys.136.195917 PMC13366088 42453997

[B73] Tan YP, Shivas RG (2024) Nomenclatural novelties. Index of Australian Fungi 31: 1–12. https://zenodo.org/records/10784072

[B74] Tanaka E, Shrestha B, Shivas RG (2020) *Commelinaceomyces*, gen. nov., for four clavicipitaceous species misplaced in *Ustilago* that infect *Commelinaceae*. Mycologia 112(3): 649–660. 10.1080/00275514.2020.1745524 32412345

[B75] Vaidya G, Lohman DJ, Meier R (2011) SequenceMatrix: concatenation software for the fast assembly of multi-gene datasets with character set and codon information. Cladistics 27: 171–180. 10.1111/j.1096-0031.2010.00329.x 34875773

[B76] Vilgalys R, Hester M (1990) Rapid genetic identification and mapping of enzymatically amplified ribosomal DNA from several *Cryptococcus* species. Journal of Bacteriology 172(8): 4238–4246. 10.1128/jb.172.8.4238-4246.1990 PMC213247 2376561

[B77] Wang WP, Bhat DJ, Yang L, Shen HW, Luo ZL (2024a) New species and records of *Pleurotheciaceae* from karst kandscapes in Yunnan Province, China. Journal of Fungi 10(8): 516. 10.3390/jof10080516 PMC11355354 39194842

[B78] Wang WP, Hyde KD, Bao DF, Wanasinghe DN, Lin CG, Shen HW, Lu YZ, Zhang ZQ, Su XJ, Li YX, Al-Otibi F, Yang LQ, Luo ZL (2024b) Lignicolous freshwater fungi from karst landscapes in Yunnan Province, China. Mycosphere 15: 6525–6640. 10.5943/mycosphere/15/1/29

[B79] Wang WP, Shen HW, Bao DF, Jeewon R, Zhang ZQ, Yang LQ, Luo ZL (2025) Biogeography and species diversity of freshwater *Savoryellomycetidae (Sordariomycetes)* fungi. Mycology 17(2): 411–480. 10.1080/21501203.2025.2509809 PMC13267038 42305155

[B80] White TJ, Bruns T, Lee S, Taylor J (1990) Amplification and direct sequencing of fungal ribosomal RNA genes for phylogenetics. PCR Protocols: a Guide to Methods and Applications. Academic Press, San Diego, 315–322. 10.1016/B978-0-12-372180-8.50042-1

[B81] Xia JW, Ma YR, Li Z, Zhang XG (2017) *Acrodictys*-like wood decay fungi from southern China, with two new families *Acrodictyaceae* and *Junewangiaceae*. Scientific Reports 7: 7888. 10.1038/s41598-017-08318-x PMC5554248 28801663

[B82] Xiao XJ, Liu NG, Ma J, Zhang LJ, Bao DF, Bai S, Al-Otibi F, Hyde KD, Lu YZ (2025) Three new asexual *Kirschsteiniothelia* species from Guizhou Province, China. MycoKeys 113: 147–168. 10.3897/mycokeys.113.139427 PMC11811712 39936083

[B83] Xiao Y, Liu JK (2025) *Xylochrysis guttulata* sp. nov., associated with *Camellia sinensis* in Sichuan Province, China. Phytotaxa 698(1): 35–42. 10.11646/phytotaxa.698.1.4

[B84] Yang J, Liu LL, Jones EBG, Hyde KD, Liu ZY, Bao DF, Liu NG, Li WL, Shen HW, Yu XD, Liu JK (2023) Freshwater fungi from karst landscapes in China and Thailand. Fungal Diversity 119: 1–212. 10.1007/s13225-023-00514-7

[B85] Yen LTH, Yamaguchi K, Hop DV, Tsurumi Y, Ando K (2021) Phylogeny and a new species of *Polylobatispora*. Mycoscience 62(3): 176–181. 10.47371/mycosci.2021.01.003 PMC9157749 37091323

[B86] Yuen T, Hyde K, Hodgkiss I (1998) Physiological growth parameters and enzyme production in tropical freshwater fungi. Material und Organismen (Berl) 32: 2–16.

[B87] Zhang H, Dong W, Hyde KD, Maharachchikumbura SSN, Hongsanan S, Bhat DJ, Al-Sadi AM, Zhang D (2017) Towards a natural classification of *Annulatascaceae*-like taxa: introducing *Atractosporales* ord. nov. and six new families. Fungal Diversity 85: 75–110. 10.1007/s13225-017-0387-z

[B88] Zhang N, Zhao S, Shen Q (2011) A six-gene phylogeny reveals the evolution of mode of infection in the rice blast fungus and allied species. Mycologia 103(6): 1267–1276. 10.3852/11-022 21642347

[B89] Zhang SN, Hyde KD, Jones EBG, Yu YD, Cheewangkoon R, Liu JK (2024) Current insights into palm fungi with emphasis on taxonomy and phylogeny. Fungal Diversity 127: 55–301. 10.1007/s13225-024-00536-9

[B90] Zhang WM, Song XY, Xie WQ, Zhou XZ, Lu J, Feng QY (2025) Morphological and multi-gene phylogenetic analyses reveal *Nigrellomyces* gen. nov. and one new species in *Pleurotheciaceae* from China. MycoKeys 122: 277–292. 10.3897/mycokeys.122.164540 PMC12464557 41018912

